# Acute Colonoscopy-Induced Splenic Rupture Presenting to the Emergency Department

**DOI:** 10.1155/2013/436874

**Published:** 2013-02-18

**Authors:** Monica D. Chow, Richard D. Shih

**Affiliations:** ^1^Robert Wood Johnson Medical School, Piscataway, NJ 08854, USA; ^2^Department of Emergency Medicine, Morristown Medical Center, 100 Madison Avenue, P.O. Box 8, Morristown, NJ 07962, USA

## Abstract

Splenic rupture due to colonoscopy is a rarely reported event in the emergency medicine literature. Patients experiencing such an occurrence are likely to report to the emergency department. This paper documents an 84-year-old female who presented to the emergency department with abdominal pain and nausea less than 24 hours following a colonoscopy. An abdominal ultrasound revealed splenomegaly and free fluid. An abdominal computed tomography was significant for a splenic laceration. She underwent radiologic guided embolization and recovered without incident. Emergency medicine physicians need to consider splenic rupture as a differential in patients presenting after colonoscopy with abdominal pain.

## 1. Introduction

Colonoscopy is considered to be a safe procedure to visualize the large intestine for various reasons including, but not limited to, colon cancer screenings, lower gastrointestinal bleeding, and inflammatory bowel disease. The most common complications are bleeding and intestinal perforation with incidence rates of approximately 1-2% and 0.1-0.2%, respectively [1, 2]. An extremely rare complication is splenic rupture, with an estimated rate of 0.004% [2]. The first published case of this complication was in 1974 by Wherry and Zehner [3]. Little has been published in the emergency medicine literature since then [3]. 

Despite the rare occurrence of splenic rupture following colonoscopy, this is a potentially life-threatening complication that may be on the rise as more colonoscopies are performed. It is therefore important that emergency physicians be aware that splenic rupture is a complication of colonoscopy and be familiar with its typical clinical presentation. 

## 2. Case Report

An 84-year-old woman presented to the emergency department (ED) with severe left upper quadrant pain, associated with nausea, which radiated into her back and left shoulder. She had a colonoscopy several hours prior to ED presentation. Her past medical history is significant for hypertension, heart disease, and hyperlipidemia. Her medications included clopidogrel, aspirin, olmesartan, atenolol, nifedipine, pravastatin, levothyroxine, and temazepam. She discontinued the clopidogrel for one week in anticipation of the colonoscopy procedure. This procedure was performed to evaluate recent symptoms of hematochezia. She had no history of prior abdominal surgery.

On ED presentation, she was diaphoretic and appeared pale. She had a temperature of 97.2 F, pulse of 50 beats/min, respiratory rate of 18 inspirations/min, and a blood pressure of 82/40 mmHg. Upon examination, there was no abdominal distention. She had normal bowel sounds and moderate tenderness in the left upper quadrant, without guarding or rebound, and no palpable masses. Her laboratory tests revealed slightly decreased sodium (130 mmol/L) and chloride (96 mmol/L) levels in addition to an increased glucose level (243 mmol/L). Her hemoglobin and hematocrit levels were both low at 9.8 g/dL and 29.3%, respectively. A chest X-ray revealed biapical pleural thickening, atherosclerotic changes of the heart, and the presence of a coronary artery stent. An abdominal X-ray did not reveal any obstruction or free air. An ED bedside ultrasound of the abdomen showed splenomegaly and free fluid in the left upper quadrant and Morrison's pouch. Following this, a contrast computed tomography (CT) of the abdomen was performed and indicated the presence of a splenic laceration of grade 3 or 4, splenic hematoma involving both intra- and extracapsular components, and extensive abdominal free fluid.

The patient initially was treated with intravenous (IV) fluids, a cardiac monitor, and oxygen. She was observed for approximately one hour. A repeat systolic blood pressure was 115 mmHg. She also received 1 unit of packed red blood cells. After reevaluation of her lab tests, vitals, and radiological results, the patient underwent successfully radiologic guided embolization of the splenic artery. She recovered uneventfully over the next several days and was discharged from the hospital without additional sequelae. 

## 3. Discussion

A review of the literature appears to indicate that splenic rupture associated with colonoscopy is a rare complication, with few reports available in the emergency medicine literature. This may be due to the fact that most cases of splenic rupture following colonoscopy are recognized immediately during the procedure and bypass the emergency department. On the other hand, many cases, like ours, can present several hours after the completion of a colonoscopy and will likely present to an ED. Being aware of this diagnosis and being able to identify the symptoms of such a patient can lead to a faster, life-saving diagnosis. Most patients with a colonoscopy-induced splenic rupture present with the typical symptoms of a trauma-induced splenic rupture within 24 hours of their colonoscopy [1–14]. However, some patients do have delayed presentation several days later [12]. There is usually abdominal pain, which is generalized or localized to the left upper quadrant [1, 2, 4–15]. Pain may also be felt in the left shoulder or left chest [4, 5, 7, 9–13, 15]. Abdominal examination generally reveals abdominal distention [1, 2, 8, 11, 13]. Vital signs and laboratory tests often indicate hypotension and/or anemia, although some patients may not have these on initial presentation [1, 2, 4, 6–13, 15]. 

 Contrast (CT) is the best tool for diagnosing a ruptured spleen after colonoscopy. Ultrasonography (US) is a useful test for diagnosing this condition, is rapid, and does not have radiation exposure and intravenous contrast risks. However, US is the best for identifying the presence of free fluid in the peritoneum and further imaging, such as a CT, is better for delineation of the extent of injury to the spleen [2, 14]. In patients that are hemodynamically stable, this test is the gold standard for the evaluation and assessment of splenic injury [1, 2, 4, 6, 8, 9, 11, 15].

 Three common treatment options for a ruptured spleen due to colonoscopy are splenectomy, splenic artery embolization, and nonoperative management. The majority of previously reported cases were managed by splenectomy [1, 2, 4, 6–8, 10–12, 14]. Patients that are hemodynamically stable can be managed successfully with conservative treatment consisting of observation, IV fluids, and blood transfusions as needed [5, 13]. However, a number of such cases will need surgical intervention [4, 7, 9, 11]. Another option, utilized in our case, is splenic artery embolization. Although only a few cases, including ours, document the use of this modality, it was a successful alternative in all reported instances and may obviate the need for surgical intervention while maintaining splenic function [2, 3, 15]. 

 An understanding of the cause behind colonoscopy-induced splenic ruptures would help to identify high-risk patients, but little information is available. Most agree that direct trauma to and/or increased traction on the spleen and/or the splenocolic ligament is a likely mechanism [1–4, 6–8, 10–15]. Risk factors for splenic laceration following a colonoscopy may include application of external pressure during the procedure, adhesions between the spleen and colon, [8, 11] and polypectomy and/or biopsy during the procedure [1–3, 6, 8, 9, 12]. Yet none of these factors was found to be common among any of the patients in the various reports. Also, complication during colonoscopy [1, 2, 6, 7, 9–14] and abnormal spleen histology [1, 4, 7–12, 14] are not risk factors. 

## 4. Conclusion

Splenic injury or rupture during colonoscopy occurs rarely but will likely present to an emergency department when it occurs. Patients typically present with abdominal pain, hypotension, and anemia within 24 hours of the procedure. ED physicians need to be cognizant of this colonoscopy complication and its presentation. 
